# Arrhythmia Care In India - Poised For The Big Leap

**Published:** 2002-01-01

**Authors:** Yash Lokhandwala

Till the late 1980s, there was precious little one could do to permanently cure arrhythmias. Cardiac surgery did not live up to its promise for ventricular tachycardia. For WPW syndrome, surgery was too invasive a procedure, with a significant chance of major complications. Electrophysiologists spent hours trying to unravel tachycardia mechanisms, and were often referred to as "electrophilosophers". Since the advent of RF ablation, this scenario underwent a sea change. Suddenly one was able to get rid of many arrhythmias with a much less invasive procedure. Electrophysiology centers mushroomed and got upgraded in the developed world.

In India, there were only a couple of centers which had tried intracardiac DC shock ablation, with varying results. In 1995, RF ablation was being performed in a handful of centers, notably AIIMS, Delhi and KEM Hospital, Mumbai. Within the next year a few more centers such as Medicity, Hyderabad, GB Pant Hospital, Delhi and Escorts Institute, Delhi also started RF ablation programmes. Today there are over 20 hospitals in various parts of the country with well-established RF ablation programmes. It is estimated that over 15,000 RF ablation procedures have been performed so far in India. This is against a backdrop of around 2 million patients with curable tachycardias in our country. Thus, there is a need for around 100 active EP centers spread all over the country. It is expected that this growth will occur over the next decade. Thus we are now past the "critical mass" and are well poised for the big leap forward.

Viewed in the context of the developing world, this is a satisfying progress. In South Asia we are today among the best equipped to offer comprehensive arrhythmia care services. This includes RF ablation, all modalities of pacing therapy, AICD implantation and also atrial fibrillation surgery. There are over 30 well-trained electrophysiologists in India and the number is steadily growing. There have been many notable academic contributions too, both in the form of original articles as well as paper presentations at peer-reviewed international fora.

Yet some deficiencies need to be looked into. The EP services are focussed in Delhi, Western Maharashtra, Hyderabad, Bangalore, Chennai, Cochin, Trivandrum and Ahmedabad. Several parts of the country, notable the East, North-East, M.P., Rajasthan, U.P. and Orissa urgently need at least a few advance centers for arrhythmia care. It is often very difficult for patients from these regions to travel and stay in far-off cities for treatment. Post-pacemaker and especially AICD follow-up and programming can be a nightmare in these settings. Hopefully this disparity will get ironed out in the coming years.

